# Shiga Toxin-Producing *Escherichia coli* and Milk Fat Globules

**DOI:** 10.3390/microorganisms10030496

**Published:** 2022-02-23

**Authors:** Arthur Bagel, Delphine Sergentet

**Affiliations:** 1 ‘Bacterial Opportunistic Pathogens and Environment’ Research Team, Université de Lyon, UMR5557 Ecologie Microbienne Lyon, CNRS (National Center of Scientific Research), VetAgro Sup, Marcy-l’Etoile, 69280 Lyon, France; arthur.bagel@vetagro-sup.fr; 2 Laboratoire d’Etudes des Microorganismes Alimentaires Pathogènes-French National Reference Laboratory for *Escherichia coli* Including Shiga Toxin-Producing *E. coli* (NRL-STEC), VetAgro Sup—Campus Vétérinaire, Université de Lyon, Marcy-l’Etoile, 69280 Lyon, France

**Keywords:** STEC, MFGM, raw milk cheese, anti-adhesion strategy

## Abstract

Shiga toxin-producing *Escherichia coli* (STEC) are zoonotic Gram-negative bacteria. While raw milk cheese consumption is healthful, contamination with pathogens such as STEC can occur due to poor hygiene practices at the farm level. STEC infections cause mild to serious symptoms in humans. The raw milk cheese-making process concentrates certain milk macromolecules such as proteins and milk fat globules (MFGs), allowing the intrinsic beneficial and pathogenic microflora to continue to thrive. MFGs are surrounded by a biological membrane, the milk fat globule membrane (MFGM), which has a globally positive health effect, including inhibition of pathogen adhesion. In this review, we provide an update on the adhesion between STEC and raw MFGs and highlight the consequences of this interaction in terms of food safety, pathogen detection, and therapeutic development.

## 1. Introduction

In the Code of Hygienic Practice for Milk and Milk Products (Codex Alimentarius, 2004), raw milk is defined as milk that has not been heated beyond 40 °C or undergone any treatment that has an equivalent effect. Raw milk is an oil-in-water type emulsion and contains almost 900 g of water and 130 g of dry matter, in varying proportions [1]. Milk is a major source of calcium, and also an important supply of proteins, for those who consume it (newborn mammals and humans). Milk contains all essential amino acids, lipids, fatty acids, vitamins, and lactose [2]. One liter of whole milk contains approximately 38 g of fat, which is present mainly in the form of milk fat globules (MFGs) [3].

Raw milk cheeses are made from raw cow, sheep, or goat milk. Different cheese-making processes can be applied to create the end-products. The various combinations of ingredients (such as milk and cultures) and cheese-making processes result in a large diversity of cheeses. No less than 1200 different cheeses are made in France [4]. These include ripened or unripened soft, semi-hard, hard, or extra-hard products, which may be coated, uncooked, or cooked pressed cheeses (with short or long ripening), blue-type cheeses, lactic cheeses, and white mold cheeses.

Raw milk is unstable due to the presence of microflora and, therefore, is often treated to optimize its conservation and to prevent microbiological hazards. The microbiota of raw milk is complex and is derived from many sources, including direct contact with the animal (teats, hides, and feces), the surface of milking equipment (particularly if hygiene practices are poor), housing, bedding, feed, air, and water [5,6,7]. On the one hand, this dynamic bacterial community plays several beneficial roles in subsequent dairy products such as facilitating fermentation and promoting the health of consumers. On the other hand, microbiota can contain spoilage or pathogenic bacteria [5]. The milk microbiota is almost eliminated during heat treatment, such as ultra-high temperature (UHT) sterilization, and microfiltration, while in raw milk the microbiota is preserved. Different studies have shown that the raw milk microbiota is useful to the human digestive system; therefore, it may be beneficial to preserve it [5,8]. In addition, the raw milk microbiota gives raw milk cheeses more varied and intense flavors than heat-treated milk cheeses. The raw milk microbiota can also influence the human intestinal microbiota, which contributes significantly to human health, for example, by modulating the immune system. The consumption of raw milk and raw milk cheeses can also reduce blood pressure in people with mild to moderate hypertension [9] and decrease allergies in infants [10,11]. However, the risk–benefit ratio of consuming raw milk products is difficult to estimate. Nevertheless, in France, it is strongly recommended to avoid giving raw milk products to children under 5 years old, pregnant women, and immunocompromised patients [12]. If poor hygiene procedures have been applied, particularly during milking, raw milk may be contaminated by pathogenic bacteria such as Shiga toxin-producing *Escherichia coli* (STEC).

STEC are foodborne zoonotic bacteria associated with large-scale epidemics that represent a major public health problem. Human STEC infection is most often linked to the ingestion of contaminated food and water, such as undercooked ground meat, raw milk cheeses, or raw vegetables. Raw milk and raw milk cheeses have been linked to foodborne infections associated with STEC in humans from different countries [13,14,15,16,17,18,19,20]. Ruminants are the primary reservoir of STEC. Milk is most often contaminated by feces (directly or indirectly) during the milking process. STEC are very frequently associated with severe forms of infection such as hemorrhagic colitis and, in very severe cases, systemic complications including hemolytic uremic syndrome (HUS). HUS is the leading cause of renal failure in children under 3 years of age. The estimated infectious dose is very low: between 5 and 50 viable cells [21,22].

The proportion of milk and dairy products involved in Rapid Alert System for Food and Feed (RASFF) notifications issued due to food contamination with STEC is very low compared to those involving meat products [23]. The RASFF is a European communication tool used when public health microbiological hazards are detected in the food chain and food products. In 2013, two RASFF notifications were related to STEC-contaminated dairy products versus 68 for meat products. In 2014, four RASFF notifications of dairy products contaminated with STEC were listed, compared with 53 for meat products. Likewise, there were 7 versus 16, 8 versus 26, and 4 versus 49 dairy-related and meat-related notifications, respectively, in 2015, 2016, and 2017 [23]. Furthermore, epidemiological studies have shown that this class of product is only a minor source of human enteric infection [24,25,26]. Interestingly, prevalence data on these enteropathogens in dairy matrices and ingestion-related outbreaks do not fit overall foodborne-related outbreak figures [26]. A study led by Douëllou et al. [27] showed that there were no differences in the key virulence properties of dairy STEC isolates compared with human isolates. The same authors hypothesized that this phenomenon might be related to an association between STEC and MFGs, thus inhibiting STEC adhesion to enterocytes.

Milk fat globules (MFGs) have a positive impact on the immune system, and their antimicrobial properties have been largely described [28,29,30,31]. The positive action of MFGs on human health seems to be carried out by the membrane (and membrane components) surrounding the globules. MFGs are small lipid droplets formed by a core of triacylglycerols (TAGs) and enveloped by a biological phospholipid triple membrane, the milk fat globule membrane (MFGM), which is derived from mammary epithelial cells [32]. The outer bilayer of the MFGM contains diverse (glyco)-proteins and (glyco)-lipids on its surface [33]. These glycoconjugates make up the glycocalyx and act as a source of specific bacterial and viral ligands [29,34].

The objective of this review is to provide an update on the interaction between STEC and raw MFGs and to highlight the implications of this interaction. First, we will review the impact of STEC in the raw milk sector. Then, we will review the mechanisms of association between STEC and fat globules. We will conclude with the beneficial and detrimental consequences of this association.

## 2. Raw Milk Sector and STEC

### 2.1. Importance of Raw Milk Cheeses

Cheeses are products with high added value and significant economic importance in France and Europe. In 2019, the annual cheese consumption per inhabitant was 26.8 kg in France and 19.1 kg in all of Europe [35]. In total, 10,630,000 t of cheese were produced in Europe in 2019 [35]. In France, cheeses represent one of the main food industries, worth approximately EUR 38.7 billion in 2017 [36]. In 2019, with all milk processing combined, French cheese production included 1,664,632 t of cow’s milk cheese, 99,265 t of goat’s milk cheese, and 59,638 t of sheep’s milk cheese [35]. The production of raw milk cheese accounted for 172,128 t of the cow’s milk cheese, 20,872 t of the sheep’s milk cheese, and 9691 t of the goat’s milk cheese produced in France in 2019 [35]. Raw milk cheeses represent the vast majority of farmhouse dairy products and approximately 75% of the volume of cheeses marketed under quality and origin identification signs (SIQO), including protected designations of origin (PDO) and protected geographical indications (PGI). Finally, a study has shown that 75% of French people consume raw milk cheeses at least once a month and that 33% of French people consume raw milk cheeses every week [37]. In contrast, no data on the worldwide consumption or production of raw milk cheeses are currently available.

Raw milk cheeses are part of French and European food heritage and are an essential tool to enhance product value and to create dynamism in our territories. They are part of a dynamic of rural development and land use planning; their production avoids the desertification of certain areas by providing a significant source of income for farmers. They are often produced under a PDO quality label. Raw milk cheeses encourage variety and diversification of production and contribute to the sustainability of rural economies. They protect traditional production areas, enhance the recognized know-how of operators, and facilitate, especially for small producers, the marketing of differentiated products with specific and clearly identifiable characteristics. In France, the competent authorities as well as scientists recognize the importance of raw milk cheese both in terms of gastronomic heritage and regional socio-economic development. At the same time, authorities and scientists seek to support the industry by improving scientific knowledge about STEC and developing methods for the control and surveillance of this bacteria from farm to fork.

### 2.2. STEC

STEC are foodborne zoonotic bacteria associated with large-scale epidemics that represent a major public health problem. Ruminants (cattle, buffalo, goats, and sheep) are the main reservoir of STEC [38,39]. Infected ruminants can be asymptomatic, harboring the bacteria in their gastrointestinal tract and shedding bacteria in their feces [40,41,42]. Detailed investigations have shown that without proper cleaning methods and udder hygiene practices, feces can contaminate the teats and udders of animals and cause milk contamination during milking [43]. When STEC-contaminated milk is used to produce raw milk cheeses, STEC can survive and be isolated in some cheeses.

**Figure 1:**
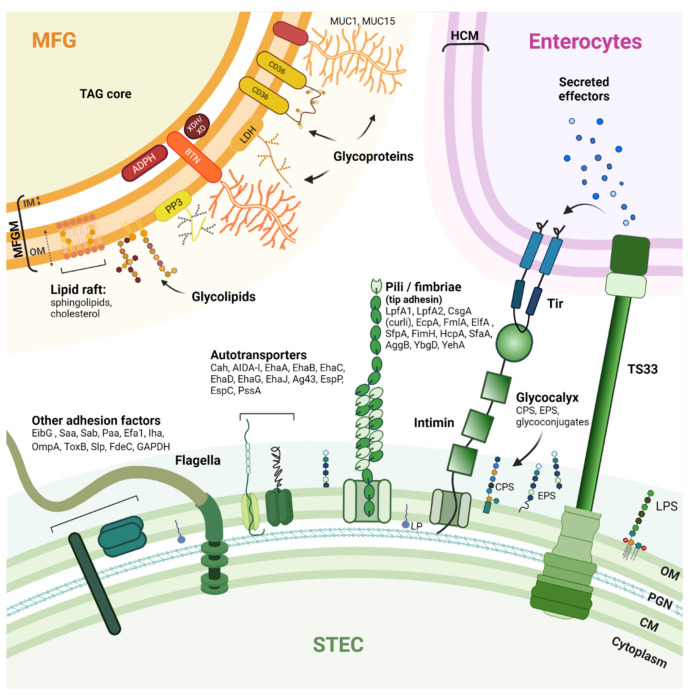
Schematic drawing of STEC adhesion factors and the bovine MFGM. STEC have an arsenal of protein structures involved in host cell adhesion. However, the adhesion mechanisms that contribute to the pathogenicity of STEC are not fully understood, and the receptors recognized by these adhesion factors are not all known. Nevertheless, some pili, autotransporters, and flagella can bind to host extracellular matrix (ECM) components such as fibronectin and laminin (glycoproteins). The MFGM is a complex trilayered structure, comprising a monolayer of polar lipids derived from endoplasmic reticulum (IM: inner membrane) and a bilayer of polar lipids originating from the apical plasma membrane of mammary secretory cells (OM: outer membrane). The structures drawn do not necessarily reflect the actual structures of the macromolecules and are not to scale. CM: Cytoplasmic membrane; PGN: Peptidoglycan; OM: Outer membrane; LPS: Lipopolysaccharide; LP: Lipoprotein; Tir: Translocated intimin receptor; T3SS: Type 3 secretion system; HCM: Host cytoplasmic membrane; CPS: Capsular polysaccharide; EPS: Extracellular polysaccharide; LpfA: Long polar fimbria subunit A; CsgA: Major curlin subunit; EcpA: *E. coli* common pilus subunit A; FmlA: Type-1 fimbria subunit A; ElfA: laminin-binding fimbria subunit A; SfpA: sorbitol-fermenting fimbria subunit A; FimH: Type 1 fimbrin D-mannose specific adhesin; HcpA: Hemorrhagic coli pilus subunit A; SfaA: S-fimbria subunit A; AggB: Aggregative adherence fimbria I subunit B; YbgD: Putative fimbria Ybg subunit A; YehA: Putative fimbria Yeh subunit A; Cah: Calcium-binding antigen 43 homologue; AIDA-I: Adhesin involved in diffuse adherence; Eha: Enterohaemorrhagic *E. coli* autotransporter; Ag43: Antigen 43; EspP: Extracellular serine protease plasmid encoded; EspC: EPEC-secreted protein C; PssA: Protease secreted by STEC; EibG: *E. coli* immunoglobulin-binding protein G; Saa: STEC autoagglutinating adhesion autotransporter; Sab: STEC autotransporter contributing to biofilm formation; Paa: porcine A/E-associated protein; Efa1: EHEC factor for adherence; Iha: IrgA homologue adhesin; OmpA: Outer membrane protein A; ToxB: Toxin B; Slp: Carbon starvation-inducible lipoprotein; FdeC: Factor adherence *E. coli*; GAPDH: Glyceraldehyde 3-phosphate dehydrogenase. MUC1/MUC15: Mucin 1/15; LDH: Lactadherin; ADPH: Adipophilin; BTN: Butyrophilin; XDH/XO: Xanthine dehydrogenase/oxidase; CD36: Cluster of differentiation 36; PP3: Proteose peptone 3; TAG: triacylglycerols.

The pathogenesis of STEC-related disease generally involves three phases: (i) ingestion of contaminated food; (ii) colonization of the intestinal epithelium by STEC; and (iii) production of Shiga toxins (Stx) that disrupt normal cellular functions and damage cells. Stx are the main virulence factors of STEC. The Stx family includes all toxins with a similar structure and biological activity. Based on their different in vitro and in vivo toxicity, amino acid sequences, or nucleotide sequences of the *stx* genes, two major types of Shiga toxins, Stx1 and Stx2, and numerous variants (Stx1a to Stx1d and Stx2a to Stx2k) have been identified [44,45,46,47]. To effectively colonize a host and cause disease, STEC have evolved mechanisms and strategies for attaching or adhering to host cells and tissues [48]. Adhesion is required so that STEC cells are not swept away by the host’s natural self-cleaning mechanisms. An arsenal of STEC surface adhesion factors have been described (Figure 1).

The ability to adhere to the intestinal epithelium and colonize the intestine undeniably contributes to the pathogenic processes of STEC cells. Thus, the vast majority of clinical isolates known to cause bloody diarrhea or HUS have one or more virulence factors that allow their adhesion to intestinal epithelial cells [44]. The major adhesion factor of clinical STEC isolates is intimin [49], a protein encoded by the *eae* gene that resides in the locus of an enterocyte effacement pathogenicity island (LEE). The pathophysiology of clinical isolates possessing the *eae* gene is characterized by the development of enterocyte attachment-effacement (A/E) lesions. These lesions are responsible for the diarrhea observed in patients [49]. Intimin attachment to the host cell requires an upstream connection of the STEC cell to the host cell cytoplasm [48]. Intimin binds to the translocated intimin receptor (Tir) protein, which is encoded by STEC and translocated into the host cell cytoplasm using a type III secretion system (T3SS) and then inserted into the host cell membrane. Although STEC isolates carrying the *eae* gene represent the vast majority of human infections, some STEC lacking the *eae* gene have been isolated from patients [50,51]. An early adhesion phase involving other adhesion factors may occur before, or in parallel with, the formation of the highly specific intimin/Tir bond. Intimin can also bind, with less specificity and strength, to certain host cell surface components such as integrin and nucleolin, and this may contribute to STEC–host cell adhesion [52,53,54]. Some studies suggest involvement of the long polar fimbriae (LPF), which recognizes moieties of eukaryotic extracellular matrix (ECM) components [55,56]. Molecular characterization studies of STEC isolates have also identified *paa, efa1, ompA, saa, sab, toxB*, and *aggR* as genes encoding virulence factors involved in adhesion [57,58,59]. Flagella are also involved in STEC adhesion by binding to mucus and mucin proteins [60]. Other proteins can interact with immunoglobulins, for example, *E. coli* immunoglobulin-binding protein (Eib) [61,62]. The complete list of virulence factors, the timing of their expression, and the mechanisms involved in STEC pathogenicity are not yet fully known. Current knowledge of STEC surface proteins is summarized in Figure 1. STEC adhesion mechanisms are further detailed in these articles: [48,59,63].

### 2.3. Milk Fat Globules

MFGs can be distinguished from other forms of fat by the milk fat globule membrane (MFGM) that surrounds a core of triacylglycerols (TAGs) (Figure 1). Complex fat supramolecular organizations are also found in egg yolk or oilseeds in the form of oleosomes [64]. The MFGM is made of phospho- and sphingolipids, cholesterol, and proteins [65,66]. As a consequence of the mechanism of milk fat secretion from mammary epithelial cells, the MFGM is a complex trilayered structure, comprising a monolayer of polar lipids derived from the endoplasmic reticulum and a bilayer of polar lipids originating from the apical plasma membrane of the mammary secretory cells [67,68,69]. The MFG size is dependent on the origin of the milk. Bovine MFGs have a mean diameter of 4 µm, while MFGs from goat (3.19 µm), camel (2.99 µm), and sheep raw milk (3.78 µm) are all smaller, and MFGs from buffalo (8.7 µm) are much larger [70]. The main MFGM (glyco)-proteins include glycoproteins mucin 1 and 15 (MUC1; MUC15), the redox enzyme xanthine dehydrogenase/oxidase (XDH/XO), butyrophilin (BTN), cluster of differentiation 36 (CD36), lactadherin (LDH); and two proteins: adipophilin (ADPH) and fatty-acid binding protein (FABP) [29,71]. For a more complete description of MFGs and MFGM, we refer the reader to these articles: [69,72,73].

## 3. STEC in Raw Milk Cheeses

### 3.1. Prevalence and Behavior of STEC in Raw Milk Cheeses

The most comprehensive studies on the prevalence of STEC in cheeses have been conducted in Europe and show that the prevalence of STEC varies from 0% to 13.1%, depending on the study [74]. In France in 2009, 2014, and 2018, surveillance plans assessed the prevalence of specific STEC isolates (*E. coli* possessing the eae and stx genes and belonging to O157:H7, O26:H11, O103:H2, O111:H8, and O145:H28 serotypes) in raw milk cheeses. These studies showed a prevalence of 0.9%, 0.2%, and 0.8% in the raw milk cheese studied in 2009, 2014, and 2018, respectively. In 2016, researchers in a French study evaluated the genetic diversity and virulence gene profiles of STEC isolated from dairy products [27]. They showed that the 197 studied isolates displayed a high genetic diversity regardless of their serotype, with Simpson’s Diversity Index ranging from 1.0 to 0.9615. In addition, their results suggested that the virulence gene profiles of the dairy isolates are a potential hazard. Nevertheless, for the isolate most frequently found in dairy products, O26:H11 STEC, gene expression was similar between human and dairy isolates except for stx1 (44% vs. 87%) and stx2 (81% vs. 23%) expression. It is important to keep in mind that Stx2 has stronger cytotoxicity than Stx1 [75]. A French team showed that during the manufacture of different types of cheese experimentally contaminated with STEC strains, there was no statistically significant strain effect for the same serotype [76,77]. However, only a few different strains were tested for each serotype. Interestingly, a serotype effect was observed in certain types of cheese. Researchers observed less growth of the serotype O157:H7 strains than of the serotype O26:H11, O103:H2, and O145:H28 strains. They hypothesized that strains belonging to serotypes O26:H11, O103:H2, and O145:H28 could be more adapted to the conditions (physicochemical parameters and microbiota) encountered in cheeses.

### 3.2. Impact of Cheese-Making Parameters on STEC and MFGs

The different processing steps applied and the origin of the raw milk used (e.g., cow, buffalo, goat, or sheep) can influence the behavior and survival of STEC [76]. The behavior of STEC (survival, growth, or inactivation) can also be influenced by temperature and by the intrinsic physicochemical properties (pH, a_w_, and % lactic acid) of, and the other microflora present in, the raw milk microbiota and added starters used in different cheeses during their manufacture. At the initial stages of cheese-making, the temperature (around 30 °C) and a_w_ value of milk provide favorable conditions for the growth of STEC and an increase in STEC level by 1–3 log CFU/g can occur [76,77]. Then, the rapid acidification (pH > 4.3) encountered during the manufacture of certain cheeses can reduce STEC cell counts by 1–4 log CFU/g, depending on the STEC serotype and the type of cheese [76,78]. Various studies have shown that when ripening is long and, therefore, the a_w_ is low, STEC numbers decrease [76]. Nevertheless, while ripening can reduce the number of STEC cells, it cannot ensure the safety of the product if the raw milk is contaminated with STEC [79,80]. The environmental conditions during cheese processing generate stress for STEC, which can affect gene expression. Although little is known about the physiological state of STEC in cheese at different stages of production, it has been shown that the cheese-making process can trigger the production of Stx phages [81].

The various milk treatments for cheese processing also impact MFG and MFGM integrity and, consequently, the molecules involved in the MFGM–bacteria association [29,82]. Such treatments include high-temperature treatments [83,84], homogenization, and enzymatic reactions [85]. One must keep in mind that for raw milk cheeses, the curd is never heated above 54 °C. Heat treatments that kill or limit bacterial growth can also damage heat-sensitive compounds, such as glycoconjugates and associated oligosaccharides, located on the MFGM surface. Carbohydrate epitopes are well-known targets of bacterial adhesion [86]. Homogenization reduces the diameter of MFGs (ranging from 0.1 to 0.5 µm [82]) and increases the total MFGM surface area available to bacteria. Furthermore, homogenization can alter MFGM composition [84,85,87]. Treatments applied during cheese production may also alter the environment and the ability of STEC to adhere to the MFGM. In addition, physical forces applied in some cheese processing (e.g., pressed cheeses) can lead to detachment of the MFGM and their dispersion either in the product or in the whey, which will be eliminated. Nevertheless, glycoconjugates may remain intact through to end-consumption in non-pasteurized dairy products.

### 3.3. Location of STEC in Raw Milk Cheeses

Douëllou et al. used epifluorescence microscopy and a specific antibody coupled to FITC to assess bovine raw milk and concluded that both STEC strains assayed were localized near MFGs [88]. A natural raw milk creaming saturation assay with STEC revealed a strain-dependent tropism for the bovine raw milk cream layer and a distinct half-saturation point (8.5–9 log_10_ CFU.mL^−1^). Similar observations were presented by Brewster and Paul, suggesting that the cream layer exhibits a high capacity for *E. coli* O157:H7, *Listeria monocytogenes*, and *Salmonella enterica* [89]. Bacterial purification from milk by creaming seems to be not species-specific but rather general to bacteria.

The discrimination of specific bacteria in a complex product, such as raw milk cheese, is a true challenge. Furthermore, due to the modifications to milk and the MFG structure that occur during the cheese-making process, bacterial localization can vary. At the macroscopic scale, Miszczycha et al. showed that in experimentally inoculated cheeses, the levels of STEC strains belonging to serotypes O103:H2 and O145:H28 were statistically similar in the core and in the rind, regardless of the ripening conditions (traditional or industrial) [76]. The levels of serotype O26:H11 were also statistically similar in the rind. Bacterial localization in raw milk cheese has also been realized by morphologic observation on electronic micrographs [90,91,92,93] or by nonspecific DNA staining and assessment by fluorescent microscopy [90,94,95]. One study observed fluorescent mCherry-tagged *Lactobacillus reuteri* in raw milk [96]. However, to the authors’ knowledge, no study focusing on the localization of STEC or other pathogenic bacteria during the cheese production process has been published.

At the microscopic scale, the localization of bacteria in other dairy products, such as fermented milk, is poorly documented. Overall, few studies have focused on the localization of STEC in dairy products. More work is needed to understand STEC behavior in raw milk cheeses and their interaction with MFGs. Based on the available literature describing other bacteria [95,97,98], STEC could potentially localize near MFGs, in serum pockets, or the protein network.

## 4. The Mechanism of STEC-MFG Association: What Do We Know?

### 4.1. General Information on Bacterial Adhesion

**Figure 2:**
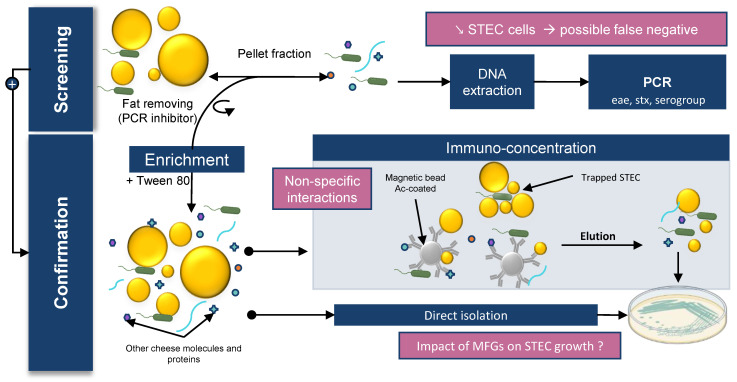
Impact of MFGs on STEC detection in dairy matrix. STEC detection in food matrices classically relies on 4 different steps: sample preparation; enrichment; detection; and confirmation by bacterial isolation.

Bacterial adhesion is a complex process involving several factors, including: (i) surface-related properties (hydrophobicity, electrical charge, roughness, and topology); (ii) cell morphological properties (size, volume, dimension, and shape); (iii) the cell surface (chemical properties, envelope type, exposed proteins, and exopolysaccharide (EPS)); and (iv) the cell’s ability to move [99]. Adhesion is a key step for bacteria (pathogenic or not), allowing colonization and growth at a host-specific site [100]. The bacterial adhesion process consists of two phases: a non-specific phase, involving physicochemical bonds, and a specific phase involving molecular factors exposed on both host and bacterial cell surface [101]. The STEC–MFG association can be seen as a host–bacteria adhesion facilitated by the origin of the MFGM and its similarities with the membrane of intestinal cells [29,68]. In this context, both biological membranes will interact together through surface components. Various glycoconjugates are anchored on the MFGM surface (Figure 2) and can act as ligands.

### 4.2. Physicochemical Interactions

Non-specific interactions have been described as the first step of adhesion, which is reversible and occurs rapidly (in the order of ~1 min) [102]. The process of initial bacterial adhesion is still not clearly understood, and physicians and microbiologists are working together to clarify the mechanisms. It is widely accepted that bacterial interaction is conducted according to the Derjaguin, Landau, Verwey, and Overbeek (DLVO) theory [103] and the extended DLVO theory [104]. The DLVO theory describes the force between charged surfaces interacting through a liquid. However, this theory may not be appropriate for modeling bacterial adhesion owing to the numerous processes involved and the influence of both biological and environmental factors (pH, ionic strength, and temperature) [105,106]. The non-specific phase of adhesion is a consequence of the balance between attractive and repulsive forces that are set up between the bacterium and the surface where it could adhere [107,108]. These forces include non-covalent interactions such as electrostatic interactions or surface charges, van der Waals forces, and Lewis acid/base interactions, as well as hydrophobic interactions [109,110]. Hydrophobic interactions and surface charges are the primary forces influencing bacterial adhesion [111].

#### 4.2.1. Cell Surface Hydrophobicity

Bacterial cell surface hydrophobicity (CSH) is probably one of the major phenomena that governs bacterial attachment to a surface [109,110]. Hydrophobic interactions are defined as the ability of two elements of similar hydrophobicity to attract each other [112]. These forces are affected by the nature of their membrane-anchored components, including amino residues that are exposed to the extracellular environment [113]. In the context of STEC and MFGs, both are surrounded by a protein-rich membrane whose anchored surface components have polar properties (e.g., proteins and phospholipids) leading to weak hydrophobic repulsions [114,115]. Interestingly, Brisson et al. showed that the adhesion of *Lactobacillus reuteri* to MFGs was strain-dependent, and the more the strain was hydrophobic, the more it adhered [94].

#### 4.2.2. Electrostatic Forces

Electrostatic forces result from the presence of a double ionic layer at the surface of a particle. The bacterial cell surface is generally negatively charged because of the carboxyl and phosphate core as well as the lipopolysaccharide (LPS) located at the surface [116]. *E. coli* surface charge is between −30 and −45 mV at milk pH [117,118]. While there are no published STEC-specific surface charge data generated with a modern instrument, some studies have shown that STEC isolates or reference strains are weakly negative [119,120]. Native MFGs are negatively charged due to the high phospholipid content of the outer layer of the MFGM [121]. The ζ-potential of native MFGs is close to −13 mV [122,123,124]. Furthermore, Malik et al. showed that the MFGM fraction could reach −20 mV at pH 6.5 [125]. When a negative surface meets another negative surface, repulsive forces are produced. Thus, in theory, MFGs and STEC should repel each other. However, it is important to note that the bacterial surface charge should be measured in an appropriate buffer that mimics the properties of raw milk, such as milk ultrafiltration permeate. There is a lack of recent experimental data with appropriate physicochemical conditions to assess the involvement of these forces in the association of STEC with MFGs.

#### 4.2.3. Van der Waals Forces

Van der Waals interactions are long-range attractive forces present in both polar and non-polar molecules and come mainly from the fluctuation of the internal charge of a particle. These forces are generally attractive and result from induced dipole interactions between molecules in a colloidal particle and a substrate [126]. Attractive van der Waals forces are ubiquitous between molecules [127] and could explain part of the interaction between bacteria and the MFGM. However, van der Waals interactions in MFGM–bacteria adhesion have not been studied.

#### 4.2.4. Lewis Acid/Base Interactions

The Lewis acid/base interaction is a polar interaction where acceptor/donor electrons enable the formation of hydrogen bonds also known as Lewis bonds [128]. This link occurs whenever ligands of strong electronegativity are associated with hydrogen. These short-range bonds are strong electrostatic interactions. Kiely and Olson showed that *L. casei* strains and MFGs behaved as electron donors and could mediate bonds [129,130]. However, the role of Lewis bonds in MFGM–bacteria adhesion was not fully investigated.

### 4.3. Specific Molecular Interactions

Bacterial molecules involved in adhesion, called adhesins, recognize specific oligosaccharide moieties or peptide residues on the surface of target cells. There are many different adhesins, including porins, complex protein structures, glycoproteins, and glycolipids. Three main types of adhesin–receptor interactions have been described: lectin–glycan; protein–protein; and hydrophobin–protein [131]. Lectins are key factors in bacterial adhesion mechanisms [132,133,134]. Lectins are adhesins that recognize glycoconjugates, the sugar epitopes generally associated with proteins or lipids. Glycoconjugates are polymeric carbohydrates composed of monosaccharides arranged in chains and preferentially present on the external leaflet either attached to lipids or proteins [135]. Commonly, the polysaccharides of glycoconjugates are referred to as the ‘glycan layer’ or ‘glycocalyx’ [136]. The glycocalyx is directly exposed to the environment, allowing interactions with other cells to facilitate cell communication, immune regulation, and adhesion [137].

A wide range of STEC isolates can be responsible for human infections, and these can be genetically different [138]. However, regardless of the strain or serogroup, STEC possess virulence factors (Figure 1) that allow attachment to intestinal epithelial cells (IECs), and these adhesion factors are generally considered essential for infection. A large range of polysaccharides exists, but only a subset is exposed at the cell surface where they can be recognized by complementary receptors. Adhesins can be found at the distal end of bacterial pili (or fimbriae). These are bacterial extracellular appendages approximately 1 to 20 μm long and <2 to 10 nm in diameter [139]. Other adhesins are anchored directly in the biological membrane of bacteria and are referred to as afimbrial adhesins [59,63].

#### 4.3.1. MFGM as a Decoy Receptor for STEC

Douëllou et al. showed that raw milk reduced the adhesion of two STEC strains (O157:H7 str. EDL933 and O26:H11 str. 21765) to intestinal cells in vitro and in vivo, whereas pasteurized milk did not [88]. Furthermore, Brewster and Paul showed that more than 98% of the pathogenic bacteria (including STEC) added to pasteurized or homogenized milk were recovered in the pellet after centrifugation, while less than 7% were recovered from raw milk, suggesting that processing could weaken the MFG–bacteria association [89]. Another study demonstrated that only MFGM proteins and glycoproteins inhibited *E. coli* adhesion in the Caco-2/HT-28 model [140]. In addition, Ross et al. suggested that the anti-infective activity of MFGM is due to the interaction of bacteria with MFGM proteins and glycoproteins rather than the interaction between MFGM and host cell receptors. In addition, modifications to MFGM surfaces such as surface roughness, zeta potential, MFG size, and phospholipid content can drastically impair the adhesive proprieties of *L. fermatum* [124]. The MFGM can also inhibit ETEC hemagglutination, suggesting that similar motifs are present on both membranes [141].

#### 4.3.2. MFGM Proteins and Glycoproteins Potentially Targeted by STEC

No published studies have focused on which MFGM proteins are recognized by STEC or which adhesins are involved. However, studies have been conducted on other bacterial models (mostly beneficial). Guerin et al. used atomic force microscopy (AFM) to show that the spaCBA pili of *L. rhamnosus* engaged with the MFGM. Another experiment conducted by Novakovic et al. demonstrated, by blot overlay, binding of the ETEC F4ac pili to various porcine MFGM or milk proteins, including lactadherin, butyrophilin, adipophilin, acyl-CoA synthetase 3, and fatty acid-binding protein 3 [142]. An extensive literature search highlighted several MFGM proteins or glycoproteins that could interact with bacteria (Table 1). As an example, Zg16 can bind peptidoglycan [143]. Milk whey proteins such as lactoferrin, β-lactoglobulin, and α-lactalbumin can be adsorbed on the MFGM by heat treatment [144,145] and can be bound by bacteria. Glycoproteins such as mucins (MUC1 and MUC15), CD59, ECM proteins (tenascin, vitronectin), butyrophilin, prolactin-inducible protein (mPIP), CD36, and alpha1-antichymotrypsin can be bacterial lectin targets (Table 1). Among this non-exhaustive list, mucins could well be potential targets for STEC. Mucins are highly glycosylated proteins known to adhere to bacteria. Mucins constitute mucus, a secreted gel that binds the intestinal microbiota and protects the epithelium from pathogens [146,147]. Additionally, EF-Tu, a ubiquitous bacterial protein that can bind many proteins and mediate adhesion, could potentially interact with the MFGM [148].

Besides proteins, a strain-specific adhesion between milk phospholipids (MPLs) and lactic acid bacteria (LAB) has been shown [195,196]. D’Incecco et al. showed that in the case of the presence of *Clostridium tyrobutyricum* spores in raw milk, these spores can be localized at the proximity of MFGs [90]. Like bacteria, the spore’s surface is decorated by polysaccharides and anchored extracellular appendages that mediate lectin–carbohydrate interactions [197,198]. However, the surface structure of *Clostridium tyrobutyricum* spores involved in the association with MFG was not identified in the study. Interestingly, further experiments conducted by D’Incecco et al. used transmission electron microscopy (TEM) to show that *C. tyrobuctyricum* interacted with the MFGM through an amorphous substance containing IgA [199].

Milk provides not only nutrients but also protection to newborns through immunocompetent cells, antimicrobial peptides, oligosaccharides, immunoglobulins (Igs), cytokines, growth factors, and lysosomes [200]. Bovine MFGs contain numerous immune-related proteins including proteins with bacterial binding capacities. Immune proteins are well characterized and known to recognize specific epitopes on pathogens. Immunoglobulins and immune cells in milk reflect the mother’s pathogen exposure and can provide immunity against some pathogens. Studies have shown that IgA, secreted-IgA (sIgA), and IgM are concentrated in the cream layer and can adsorb onto human [201,202] or bovine [90] MFGM surfaces. These adsorbed Igs may act as mediators of bacterial adherence to MFGs. Other studies have demonstrated the efficacy of bovine Igs against various human pathogens related to diarrhea [203,204,205]. Antibodies against pathogenic *E. coli* are common in samples of human milk [206,207]. Several studies have also shown that bovine colostrum contains antibodies to *E. coli* O157:H7 and other pathogens, regardless of whether the animals were immunized (vaccinated) or not. These antibodies can confer protection against relevant pathogens to humans [208,209,210]. Oliveira et al. showed that Igs could interact with ETEC fimbrial proteins and block adhesion to host receptors [211]. It has also been reported that K88-positive *E. coli* adhere to MFGs through IgA [212].

The spontaneous agglutination of MFGs in cold milk is due to the presence of immunoglobulins, called cryoglobulins [213]. Cryoglobulins are large molecules that precipitate at low temperatures (<37 °C) and disperse again on warming. Cryoglobulins are probably involved in bacterial clarification during natural creaming [214]. Immunoglobulin cell receptors are present on both the bacterial surface and MFGMs and, therefore, could act as a bridge. A generic IgG receptor is present in cold-stored MFGM preparations, but bacterial interaction has not been shown [215]. The polymeric immunoglobulin receptor (pIgR) is present on intestinal epithelial cells and facilitates the transcytosis of Igs, especially IgA, and immune complexes [177,216].

Toll-like receptors 2 and 4 (TLR2 and TLR4), which recognize foreign antigens, are present at low levels on MFGMs [33]. For example, FimH, the adhesive tip from the Type 1 fimbriae of *E. coli*, binds to mannose, TLR4, and CD48 [183,217]. Furthermore, TLR2 recognizes lipoteichoic acid (LTA), peptidoglycan, lipoprotein, curli, and other pathogen-associated molecular patterns (PAMPs) [184,185,218]. CD36 is a scavenger receptor that binds lipopolysaccharide (LPS) and other ligands [219]. Cathelicidins are antimicrobial peptides that can bind LPS [157]. Peptidoglycan recognition protein 1 (PRP1) is an antibacterial protein that can kill Gram-positive bacteria by binding to peptidoglycans and interfering with peptidoglycan biosynthesis [180].

## 5. Consequences of the STEC–MFG Association

### 5.1. Difficulties in Detecting STEC in Raw Milk Products

STEC detection in food matrices classically relies on four different steps: sample preparation; enrichment; detection; and confirmation by bacterial isolation. The enrichment step consists of adding an enrichment broth to the matrix to enable growth of the target bacteria. In the detection step, a genetic method is implemented to detect the presence of target bacteria by PCR screening. Finally, the confirmation step is carried out. This confirmation is based on isolation of target bacteria grown on selective media. Immunoconcentration tools using magnetic beads spiked with antibodies can also be used in this step. The ISO TS13136:2012 is the standard currently used to detect and isolate STEC belonging to O157, O26, O103, O111, and O145 serogroups and carrying *eae* and *stx* genes in food samples.

STEC detection in raw milk cheeses is particularly challenging. First, bacterial DNA is extracted from a specific volume of enrichment broth. Then, STEC target genes (*eae*, *stx,* and genes encoding one of the five somatic antigens) are detected. However, the microflora of cheese contains bacteria that carry some of the genes used to screen for STEC. For example, some non-STEC strains of *E. coli* (such as enteropathogenic *E. coli* (EPEC)) carry the *eae* gene or contain phages carrying the *stx* gene. Some other bacterial strains can also carry the *stx* gene, including *Citrobacter freundi*, *Shigella* spp., *Acinetobacter*, *Aeromonas* spp., *Hafnia alvei*, *Escherichia albertii*, *Escherichia cloacae,* and enterotoxigenic *E. coli* (ETEC) [44].The presence of these bacteria in raw milk cheese can lead to positive PCR results even though STEC isolates are not present in the enrichment broth (false positives). Furthermore, the performance of the methods (LOD) varies depending on the methods used and the matrices analyzed.

In general, STEC detection is more difficult in cheese than in meat. The LODs of the different detection methods are approximately 5–10 cells/g and 10–50 cells/g for bovine meat and raw milk cheese, respectively [220,221]. The presence of a richer flora and a higher amount of fat in cheese compared with meat may be an explanation. As discussed above, bacteria are preferentially found in contact with MFGs in raw milk products; however, the available detection kits perform DNA extraction on the pelleted fraction. Moreover, MFGs can interfere with DNA extraction methods by blocking spin column filters and acting as a PCR inhibitor [222,223]. Lower efficiency of bacterial DNA extraction can lead to false negative PCR results from enrichment broth samples. Several authors have described this phenomenon for various milk origins and suggest performing the extraction on both the cream and pelleted fraction. Sun et al. showed that cream harbors bacterial species that may be underestimated when skimmed milk, rather than whole milk, is used for DNA extraction [96]. Stinson et al. showed that a significant amount of human and bacterial cells remains with the cream and that bacterial DNA profiles can vary between fractions, especially for staphylococcal species [223]. The authors suggested that high-speed centrifugation may be insufficient to pellet bacterial or eukaryotic cells from milk. Furthermore, MFGs and proteins such as caseins can disrupt the interaction between immunomagnetic beads and STEC during the confirmation step. Tween 20 can be added at this stage to improve sample homogenization and block non-specific interactions [224].

Finally, STEC isolation to confirm the presence of the bacteria is also very difficult in raw milk cheese because the cheese microbiota limits STEC growth on agar plates. In addition, the different challenges encountered during cheese processing as well as the stresses suffered by STEC during the detection protocol can lead to viable but nonculturable (VBNC) isolates [225].

These studies emphasize the importance of using whole milk instead of skimmed milk for DNA extraction, but MFGs can perturb downstream applications. To improve the recovery rate of STEC in raw milk products, it seems essential to identify the nature of the STEC–MFG association in order to dissociate the two before performing the detection process. The identification of milk components involved in PCR inhibition and the improvement of DNA purification methods would allow the development of new kits to extract bacterial DNA from milk and cream. It should be noted that, despite these limitations, available DNA kits are still very effective. Quigley et al. showed that commercial kits provided very pure DNA suitable for PCR amplification from raw milk and raw milk cheese [226]. Furthermore, ISO 6887 recommends the addition of Tween 80 when enriching high-fat matrix to improve detection and bacterial isolation. However, no formal study has shown a significant effect due to the addition of Tween 80. Finally, at the enrichment temperature (37 °C or 41.5 °C), the lipids form a surface layer that may contain the desired bacterial cells and contribute to the reduction of available oxygen and thus modify the physiological state of the bacteria.

Figure 2 brings together the different concepts discussed in this section.

### 5.2. Impact of Creaming on the Presence of STEC in Milk

One of the industry’s goals should be a non-invasive method to eliminate pathogenic bacteria in raw milk without affecting the nutritional qualities and raw milk microbiota of the final product. Many techniques exist, such as bactofugation and microfiltration, [227], but these techniques affect MFG structure [82] and also remove the raw milk microflora. In our lab, we performed a raw milk skimming assay by electric centrifuge, and *E. coli* were not found in significant numbers in the cream fraction [228]. Stronger centrifugal forces are applied by the centrifuge; therefore, the STEC–MFG association is probably too weak to overcome the centrifugal forces. However, it was reported that natural creaming produces reduced-fat milk with a lower bacterial count and fewer somatic cells [229]. MFGs spontaneously rise to the surface due to the difference in density between MFGs and the aqueous phase (Stokes’ law) [230]. As previously discussed, STEC were predominantly found in the cream layer after raw milk creaming [88]. Therefore, performing natural creaming methods before cheese transformation could decrease the level of STEC in the final product. However, no study has been conducted in experimental field conditions (with low STEC contamination levels).

### 5.3. Anti-Adhesive Strategies

As bacterial adhesion is the first step of infection, inhibiting this step is a key strategy for infection control. Competition for the natural binding sites of pathogenic bacteria by mimetic receptors could inhibit pathogen attachment. Several natural food components could act as efficient inhibitors of pathogen adherence [30,100,231,232,233], especially milk components [29,234]. Moreover, numerous experimental studies have shown that the association of bacteria with MFGs could prevent the adhesion of several enteropathogens to enterocytes through mimetic receptors [142,172,235,236,237,238,239,240]. To avoid STEC adhesion to the epithelium of the intestinal mucosa, the STEC–MFG complex must be maintained at the site of STEC adhesion. Therefore, the expression of STEC genes involved in adhesion must be able to occur in product and during the human digestive process.

MFGM glycoconjugates are the main macromolecules involved in the anti-adhesive properties of milk against enteropathogens [29,73]. The MFGM protein fraction shows similarities to intestinal cells. Major MFGM proteins are conserved between species, although there are variations in protein expression levels and molecular functions between species and stages of lactation [241]. The MFGM was recently recognized as a high value-added ingredient, and the valorization of this by-product is constantly increasing. The MFGM, or some of its components, are added to infant milk formulas because of the MFGM’s beneficial properties [241,242,243,244,245]. More studies should be conducted to identify the MFGM surface glyco-epitopes recognized by STEC. These data could lead to pharmaceutical development of a specific drug to treat STEC infections. Currently available therapeutic solutions for STEC are controversial.

## 6. Conclusion and Future Directions

**Figure 3:**
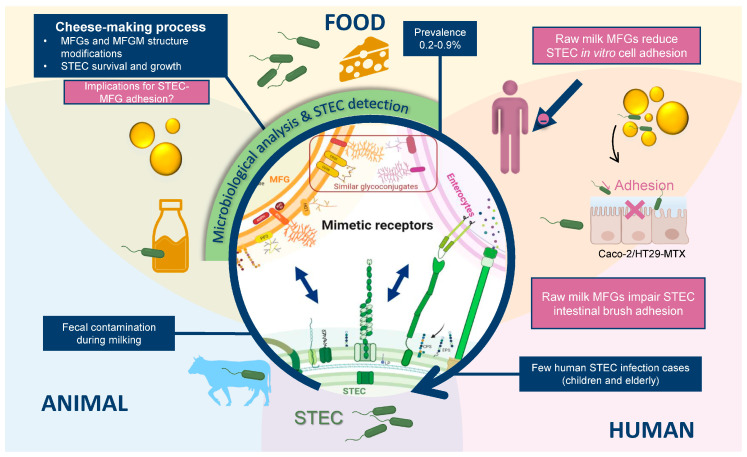
MFGM as an anti-adhesive modulator of STEC. Ruminants (cattle, buffalo, goats, and sheep) are the main reservoir of STEC. Infected ruminants harbor the bacteria in their gastrointestinal tract without any symptoms of illness and shed them in their feces. Raw milk can reduce the adhesion of STEC strains to host intestinal cells. The association of STEC with MFGs can be seen as a host–bacteria adhesion facilitated by the epithelial origin of the MFGM and its similarities with the membrane of intestinal cells. Both biological membranes interact through surface components. Various glycoconjugates are anchored on the MFGM surface and can act as ligands. The molecules involved in the association have not been identified.

MFGs can associate with beneficial and pathogenic bacteria, including STEC. To date, we do not have sufficient evidence to confirm the adhesion of STEC to bovine MFGs and more specifically to the MFGM. However, the research discussed in this review highlights a real association between STEC and MFGs that impacts bacterial pathogenicity (Figure 3). Based on studies with other bacterial models, STEC adhesion factors may adhere to MFGM glycoconjugates, resulting in impaired bacterial adhesion to host cells. The association mechanisms remain unclear, but several phenomena are likely to be involved. The large diversity of STEC isolates and the complexity of bacterial adhesion make it difficult to study these mechanisms. Few studies have assessed STEC adhesion to the glycoconjugates contained in raw milk products. We need to better understand the mechanisms of this adhesion, including the molecular factors involved and the binding strength. Therefore, it is important to identify and better characterize STEC adhesion factors and their implication in the infection cycle. In addition, more complete studies should be conducted to elucidate the location of this pathogen in raw milk cheese products and how the STEC–MFG association changes once the product is ingested by humans. To ensure inhibition of STEC adhesion to enterocytes, the STEC–MFG complex must be resistant to human digestive processes.

Research on the anti-adhesive properties of bovine MFGM components is recent and needs to be further pursued as a new source of antibacterial molecules. In the long-term, specific (glyco)-proteins derived from the MFGM could be developed as preventive or therapeutic tools against STEC or other enterobacteria. Nevertheless, bacteria–MFG adhesion is not specific to STEC and can lead to contrasting effects depending on the bacterial species involved. For STEC or other pathogenic bacteria, the association has an overall health benefit by reducing bacterial adhesion to host cells. In contrast, reducing the adhesion of beneficial bacteria or probiotics may not be as beneficial. Parallel studies using various bacterial models would improve our understanding of this phenomenon and may help to identify a molecule that inhibits unwanted adhesions, while preserving beneficial microflora adhesion. In addition, anti-adhesive molecule(s) could be used to improve STEC detection in dairy matrices. MFGs can affect the detection process and decrease the chances of isolating bacteria from a suspect product. Finally, the influence of the interaction of dairy matrix and specific components such as MFGM on the regulation of STEC virulence genes should be studied.

## Figures and Tables

**Table 1:** MFGM proteins or glycoproteins that are potentially bound by STEC.

Bovine MFGM Components	Bacterial Components	References
Adipophilin * (ADPH)	F4ac (*E. coli*) fimbria	[142]
Alpha 1-antichymotrypsin (serpin)	-	[149]
Annexins A1, A2, A5	LPS (lipid A), OmpB, YadC (tip adhesin of Yad fimbriae)	[150,151,152]
Apolipoprotein serum amyloid A protein	OmpA	[153]
Apolipoproteins	LPS	[154,155]
Butyrophilin *	F4ac (*E. coli*) fimbria	[29,142]
Calnexin	LPS, peptidoglycan	[156]
Cathelicidin 1	LPS, LTA	[157]
CD36 *	LPS, LTA	[29,158]
CD5L protein	-	[159]
Elongation factor thermal unstable Tu (EF-Tu)	-	[148]
Fatty acid-binding protein *	F4ac (*E. coli*) fimbria	[142]
Fibrinogen	Fibrinogen-binding protein (MSCRAMMs), curli	[160,161,162,163,164,165]
Galectin 7	LPS	[166]
Gelsolin	LPS, LTA	[167]
Immunoglobulins	Many bacterial proteins	-
Integrin	Many bacterial proteins	[52,165,168,169,170,171]
Lactadherin *	F4ac (*E. coli*) fimbria	[142,172]
Lactoferrin	OMPs	[173]
Macrophage scavenger receptor	LPS, LTA	[174]
MUC1 *, MUC15 *	Many bacterial proteins	[175]
Polymeric immunoglobulin receptor (PIgR)	Ig-mediated adhesion, direction interaction via adhesin	[176,177]
Prolactin-inducible protein (mPIP)	-	[178,179]
Peptidoglycan recognition protein 1	-	[180]
Protein disulfide-isomerase (PDI)	-	[181]
Toll-like receptor 4, 2	Many bacterial proteins	[182,183,184,185]
Uromodulin	Surface layer protein A, FimH	[186,187]
Vimentin	Many bacterial proteins	[188]
Vitronectin	Many bacterial proteins	[189]
Zymogen granule protein 16 homolog B	LTA, peptidoglycan	[190]
β-lactoglobulin	Spa pili	[191,192]

MFGM proteins were obtained from [33,193,194]. * Major MFGM proteins.

## Data Availability

Not applicable.
